# Predicting the Effect of Mould Material and Design on the Efficiency of the LLDPE Rotational Moulding Process: Thermal and Time Analyses

**DOI:** 10.3390/ma19020253

**Published:** 2026-01-08

**Authors:** Karolina Głogowska, Janusz Wojciech Sikora

**Affiliations:** Department of Technology and Polymer Processing, Faculty of Mechanical Engineering, Lublin University of Technology, Nadbystrzycka 36, 20-618 Lublin, Poland; janusz.sikora@pollub.pl

**Keywords:** rotational moulding, linear low-density polyethylene (LLDPE), computer simulation (RotoSim), mould material, mould wall thickness, heating chamber temperature

## Abstract

This study evaluates the effect of mould material and mould wall thickness on the thermal behaviour and cycle time of the LLDPE rotational moulding process by using RotoSim-based numerical simulation. This study was performed using three different metallic materials (low-carbon steel, brass, and aluminium), mould wall thicknesses of 3, 5, and 8 mm, and oven temperatures of 230, 250, and 270 °C. The simulations demonstrate that both mould material and wall thickness significantly influence the temperature evolution in the mould cavity and the overall cycle duration. Aluminium moulds provided the mould-cavity temperature closest to the oven conditions, the longest time with the polymer in the plastic/molten state, and the shortest total cycle time compared with steel and brass moulds. Increasing the mould wall thickness prolonged the cycle time for all materials, with the extension occurring primarily during the cooling stage. For a 3 mm wall thickness at 230 °C, the shortest cycle time was 2900 s (Al/3/230) and the longest was 3300 s (S/3/230). For an 8 mm wall thickness at 270 °C, the shortest cycle time was 4400 s (Al/8/270) and the longest was 4900 s (S/8/270). These results indicate that selecting an appropriate mould material and wall thickness can be an effective approach to shortening the cycle time and improving the efficiency of LLDPE rotational moulding.

## 1. Introduction

Rotational moulding is one of the few thermoplastic processing techniques that do not require the use of complex and expensive processing tools. In addition, this technique allows for a relatively inexpensive production of high-volume products. Rotational moulding is predominantly used for the production of hollow parts [1,2], usually of large size, whose wall thickness can be adjusted by changing the ratio of mould speed about the main and auxiliary axes [3,4]. Rotationally moulded polymer products constitute a relatively small share in the world’s production of finished products (about 0.7%) [5]. Only about 2500 companies worldwide use this method to manufacture finished parts.

Although rotational moulding has been in use for more than 50 years, it is only in the last decade that this technique has been significantly developed, thus becoming an alternative to injection and blow moulding as well as vacuum-forming processes for polymers [6].

Moulds used in rotational moulding do not have to be resistant to elevated pressure. Therefore, they are usually thin-walled structures that should primarily be characterised by high thermal conductivity. Rapid heat transfer into and out of the mould cavity is desired. In most cases, the material of a mould is selected based on the complexity and size of a moulded part and the cooling method. The mould should exhibit sufficient strength and rigidity to carry its own weight and that of a moulded part [1]. A vast majority of moulds used in rotational moulding are made of different metals and their grades: e.g., aluminium [7,8,9,10,11,12], low-carbon steel [8,10,13], stainless steel [8,10,14,15], sheet steel [13,16,17], brass [10], and copper–nickel [18]. In contrast, the mould wall thickness typically ranges from 2 to 10 mm and varies depending on the mould construction material [8,19]. There is no clear, universally established relationship for selecting a mould wall thickness across the different metallic mould materials analysed in the literature.

A selection of thermal properties of mould materials is given in Table 1. Previous studies do not provide detailed information on the specifications of mould materials; they are limited to providing general definitions of the materials and their heat transfer coefficients. Thermal properties data are essential for the correct modelling of any heat transfer process.

The selection of mould material depends on the part dimensions, the required detail precision, tool durability, and the production volume. Below, practical applications for the most commonly used mould types are presented.

Sheet steel moulds are used mainly for large parts with simple geometry, where high durability and mechanical resistance are required [1]. Examples include industrial tanks of up to 50,000 litres, inspection chambers, and sewer/manhole components. They are relatively lightweight compared to massive cast moulds, which facilitates handling on rotational moulding machines. Aluminium moulds (cast or CNC-machined) dominate in production due to their excellent thermal conductivity, which shortens the heating and cooling stages. They are used for complex shapes, such as kayaks, boats, chemical tanks, garden planters, or housings with functional details (threads, undercuts) [20]. They are well-suited to short and medium production runs, such as, for example, insulated containers or toys. Brass moulds are less commonly used in rotational moulding, but they can be applied in prototyping and small series that require a high-quality surface finish due to their ease of machining and corrosion resistance.

Aside from selecting the mould material, heat transfer conditions in rotational moulding can also be modified by increasing the thermal conductivity of the polymer charge by using thermally conductive fillers. The literature highlights that polymer-based composites can improve heat transport when a suitable polymer matrix is combined with conductive fillers, although the effectiveness depends on factors such as filler dispersion and polymer–filler interfacial thermal resistance [21]. In rotational moulding, graphene-enriched LLDPE has been reported to exhibit increased thermal conductivity and process energy savings, which were attributed to enhanced heat transfer during processing [22]. In the present work, however, neat LLDPE was used, and the analysis was intentionally limited to the influence of metallic mould material and geometry; incorporating graphene-filled LLDPE is, therefore, proposed as a future extension.

With the development of computer technology, more and more complex problems of polymer processing are being solved. Computer systems for modelling the processing of polymeric materials can be divided into:

- General-purpose systems, e.g., ANSYS Polyflow [23] or ANSYS Fluent [24].
- Special-purpose systems that are designed to simulate specific processes, such as rotational moulding-RotoSim [25]; extrusion, e.g., NEXTRUCAD [26], REX [27], AKRO-CO-TWIN [28]; injection moulding, e.g., AUTODESK Moldflow [29] or Moldex3D [30].

RotoSim is a program that is used for modelling rotational moulding processes. It is based on a complex mathematical model of physical processes occurring during rotational moulding. The program is equipped with a number of functions, allowing for an accurate reproduction of the real rotational moulding process by the simulated one. The RotoSim software (Belfast, Great Britain) was developed by the researchers from Queen’s University in Belfast, R.J. Crawford and P. Nugent [31]. The specialist computer software for modelling rotational moulding makes it possible to predict the stability of this process based on technological conditions, geometrical data and mould properties, and the machine type, as well as the properties of the polymer being processed. The system is useful not only as a training package, but it also provides practical data on mould design and optimal processing machine settings [31]. Although rotational moulding is not a complex process in practice, it is characterised by many complex interactions, such as heat transfer, polymer state transitions, and biaxial rotation of the mould, which makes this process difficult to investigate and simulate. The existing literature on computer simulations of rotational moulding is not extensive [7,31,32,33].

The use of RotoSim in research works highlights the significant role of advanced simulation tools in the development and improvement of manufacturing processes. Not only do computer simulations help determine optimal process parameters, but they also enable the exploration of new methods and techniques to improve production, while minimising the risks and costs associated with physical experiments [34]. The use of RotoSim makes it possible to understand the complex thermal phenomena and phase changes occurring in materials during rotational moulding, which directly affects the quality of finished products.

The specific nature of rotational moulding makes it difficult to obtain moulded parts with satisfactory functional and operational properties. The literature review has revealed a need for a study investigating the impact of variable factors on the rotational moulding process and moulded part quality. Given the lack of detailed research works investigating the effects of the type and wall thickness of metallic materials, heating chamber temperature, and heating cycle duration on rotational moulding, the present study will fill this research gap and provide a more detailed insight into the mechanisms of rotational moulding. Hence, this paper presents the effects of selected mould materials, mould wall thicknesses, mould temperatures, and heating times of the mould in the heating chamber on the rotational moulding process.

In this study, low-carbon steel, aluminium, and brass were selected because they represent materials that are realistically used for manufacturing moulds and, at the same time, provide a clear contrast in thermal conductivity and specific heat capacity. This allows us to assess to what extent differences in heat transfer through the mould wall translate into the mould-cavity temperature and the duration of individual stages of the cycle. The mould wall thicknesses of 3, 5, and 8 mm were chosen as representative values for thin-walled and medium/thick mould constructions encountered in practice, reflecting the trade-off between mould stiffness/durability and the mould heating and cooling times.

The aim of this work is to quantitatively evaluate the effect of metallic mould material and mould wall thickness on the thermal/time evolution of LLDPE rotational moulding using numerical analysis (RotoSim) under conditions typical for the process. The results provide a basis for selecting mould material and geometry to shorten the cycle time and improve process efficiency.

## 2. Materials and Methods

Experiments were conducted on the samples of linear low-density polyethylene (LLDPE) in powder form, with the trade name DOWLEX^®^ 2629UE. This LLDPE is widely used in rotational moulding [6]. The polymer is manufactured by Dow Chemical Company (Midland, MI, USA) [35]. The basic specifications of this material are listed in Table 2.

According to the DSC (Differential Scanning Calorimetry) data for LLDPE used in rotational moulding, the characteristic transition temperatures are the following: the melting temperature *Tm* = 124 °C, the glass transition temperature *Tg* = −123 °C to −125 °C, and the crystallisation temperature Tc = 110 °C [3]. In the rotational moulding process, Tm and Tc are the key technologically relevant temperatures because they correspond to polymer melting and powder–particle fusing during the heating stage, as well as to solidification/crystallisation during cooling. These phenomena directly shape the temperature profile and the cycle time. Tg does not significantly influence the process characteristics analysed here, since it lies far below the processing temperature range and is not reached within the considered processing window. At the same time, it should be emphasised that Tg defines the boundary between brittle and ductile fracture behaviour in thermoplastics (fracture below *Tg* is typically brittle, whereas above *Tg*, it is more ductile), while fracture toughness and the fracture mechanism also depend on the temperature, environment, loading rate, material composition, and microstructure, together with geometric effects.

According to the manufacturer, this plastic is designed for the production of films by extrusion blow moulding, injection moulding, and rotational moulding. Given its good processing and functional properties, as well as low production costs, LLDPE is widely used primarily for the manufacture of intermediate bulk containers, small tanks, drums for chemicals, boats, fish crates, etc. [35].

Following a review of the literature and drawing on our previous experience, three metallic materials were selected for mould design. These were as follows: low-carbon steel, brass, and aluminium.

Low-carbon steel and aluminium moulds had the following wall thicknesses: 3 mm, 5 mm, and 8 mm. The brass mould, on the other hand, had a wall thickness of 3 mm and 5 mm. In Table 3, the chemical compositions and properties of the mould materials used in this study are given.

The moulds were made of the following materials:

- Low-carbon steel DC01 (1.0330);
- Brass CW508L (CuZn37);
- Aluminium AlMg3 (AW-5754);

The inside corners of the mould cavity were rounded. According to R. J. Crawford, the rounded corners of the mould cavity reduce stress concentrations, and they facilitate the distribution of polymer and the manufacture of a moulded part with a uniform wall thickness [8].

Experiments were preceded by numerical simulations using the finite element method. A theoretical analysis of the rotational moulding process for linear low-density polyethylene using moulds made of different metallic materials was performed in the RotoSim program (Version 12). The primary objective of the simulations was to investigate the impact of the applied variable factors on the rotational moulding of linear low-density polyethylene.

The simulations made it possible to determine the effects of the mould material, mould wall thickness, temperature, and heating time in the heating chamber on the rotational moulding process, as well as the distribution of temperature during the entire process, cycle time, and the rate of internal air temperature increase.

The first stage of the study involved designing a 3D solid model of a moulded part using the Solid Edge 2022 software from SIMENS (Plano, TX, United States) and saving it as an STL. file. The model consisted of 3658 triangular finite elements and was entered into RotoSim. The shape and dimensions of the numerical moulded part were the same as those of the experimental one.

Prior to the simulations, constant and variable factors corresponding to the actual rotational moulding process were established. A machine (Institute for Engineering of Polymer Materials and Dyes, Toruń, Poland) with a biaxial rotating mould was selected from a database of processing machines. Specifications of the processing machine were entered into the Machine Data module in accordance with the technical characteristics of a laboratory rotational moulding machine. The properties of the linear low-density polyethylene DOWLEX^®^ 2629UE, as given by the manufacturer and listed in Table 4, were entered into the Polymer Data module. The properties of the tested mould materials, i.e., low-carbon steel, brass, and aluminium, were taken from the Mould Material Data module.

Values of the variable factors applied in the experiments are listed in Table 4.

## 3. Results

### Numerical Results of Rotational Moulding

This section reports the results of the computer simulations. The results are presented as plots. It should be stressed that for experimental validation purposes, the constant and variable factors were the same as those applied in the real rotational moulding process.

General characteristics of the rotational moulding process are given in plots 1–3. These plots show the temperature on the outer surface of the mould, peak internal air temperature, and the temperature of the LLDPE powder during the rotational moulding cycle. The above results were obtained for moulds made of low-carbon steel, brass, and aluminium, with their wall thicknesses of 3 mm and 8 mm. The numerical results of rotational moulding are presented for two extreme temperatures in the heating chamber, i.e., 230 °C and 270 °C. The numerical results of peak internal air temperature and cycle time were validated experimentally. For comparison purposes, all plots were made in the same scale. The plots show the peak internal air temperature because, according to researchers, it is one of the most important factors affecting the rotational moulding process and properties of moulded parts [10].

**Figure 1:**
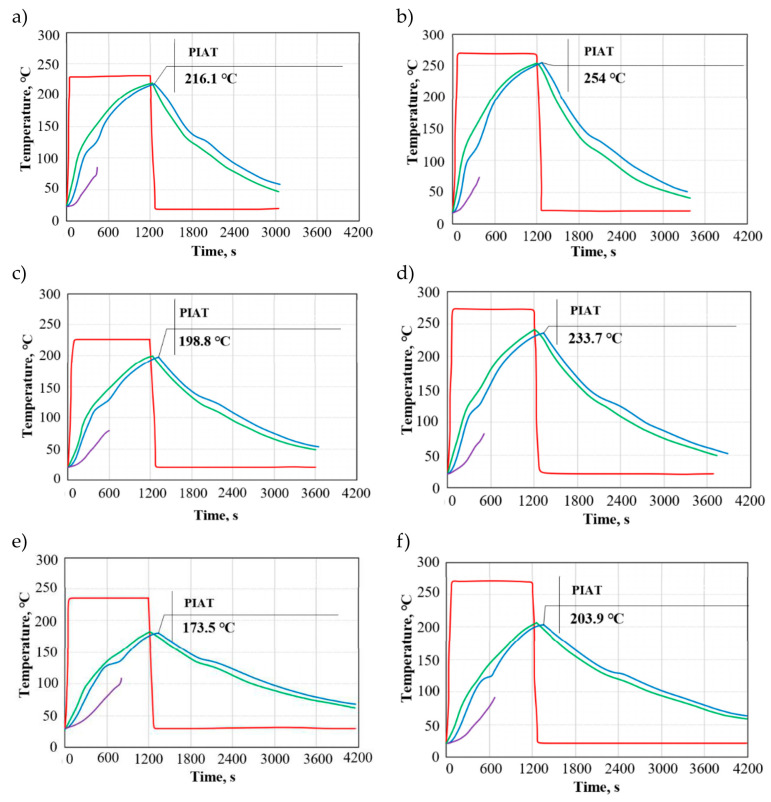
Temperatures during a rotational moulding process conducted with low-carbon steel moulds: (**a**) S/3/230, (**b**) S/3/270, (**c**) S/5/230, (**d**) S/5/270, (**e**) S/8/230, and (**f**) S/8/270; where: red curve—temperature in the heating chamber, green curve—temperature on the mould surface, blue curve—peak internal air temperature (PIAT), violet curve—temperature of the polymer.

Figure 1a–f shows the distribution of temperature as a function of time for low-carbon steel moulds. Each analysed case is characterised by a different pattern of temperature distribution on the mould surface, internal air temperature, and the temperature of the polymer. The temperature distribution depends on the applied temperature in the heating chamber and the wall thickness of the mould. In the plots, one can observe a difference between the temperature in the heating chamber and the internal air temperature. This difference becomes more pronounced as the wall thickness of the moulded part is increased.

At first, the temperature of the mould wall and that inside the mould cavity increase rapidly; this increase becomes slower as LLDPE adheres to the inner surface of the mould cavity and mould wall thickness increases. Once the cooling cycle begins, the temperature on the mould surface and the internal air temperature decrease slowly. The tool system cooling is initially rapid due to a large temperature difference between the mould and the cooling medium. After solidification, the cooling proceeds steadily until reaching the temperature at which the rotational moulding cycle is completed.

The peak internal air temperature (PIAT) values were 216.1 °C (S/3/230) and 254 °C (S/3/270), while the lowest ones were 173.5 °C (S/8/230) and 203 °C (S/8/270). An increase in mould wall thickness reduces the peak internal air temperature by about 6% (3 mm), 14% (5 mm), and 24% (8 mm) relative to the heating chamber temperature, for 230 °C and 270 °C alike. In addition to the heating chamber temperature and mould wall thickness, the peak internal air temperature depends on the thermal properties of low-carbon steel, such as thermal conductivity and thermal diffusivity.

Below are the values of the peak internal air temperature increase during the heating cycle for the different configurations of variable factors: S/3/230—10.80 °C, S/3/270—12.70 °C, S/5/230—9.94 °C, S/5/270—11.68 °C, S/8/230—8.67 °C, and S/8/270—10.20 °C.

The above results show that the internal air temperature increased depending on the heating chamber temperature (230 °C, 270 °C) and wall thickness, with this increase being 17.59% (3 mm), 17.51% (5 mm), and 17.65% (8 mm), respectively. For a higher temperature in the heating chamber (270 °C), the rate of internal air temperature increase was higher than that observed at a lower temperature (230 °C). For the same temperature in the heating chamber, the rate of the internal air temperature increase decreased as the mould wall thickness increased.

Throughout the entire cycle of the rotational moulding process, heat is transferred between the mould wall, polymer (for various states of aggregation), and the environment (air inside the heating chamber and mould cavity). The transfer of heat to the polymer occurs by convection due to a lack of perfect contact between the mould surface and the LLDPE powder, which results from biaxial rotation of the tool system. As a result, the temperature of the mould and that of the powder at contact points are not the same. The LLDPE powder particles heat up by conduction when the heat is transferred through the mould walls from the heating chamber to the mould cavity and thus to the polymer [36]. A mould with a smaller wall thickness has a reduced resistance to heat conduction through the wall to the polymer, which affects the rate of its transition from a solid to a liquid/plastic state.

**Figure 2:**
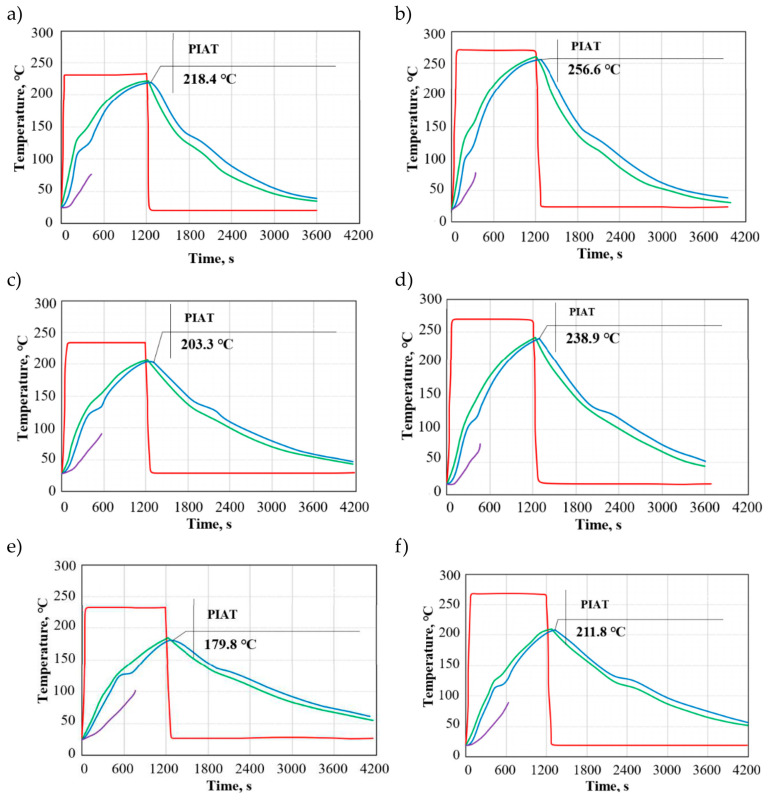
Temperatures during a rotational moulding process conducted with brass moulds: (**a**) M/3/230, (**b**) M/3/270, (**c**) M/5/230, (**d**) M/5/270, (**e**) M/8/230, and (**f**) M/8/270, where the red curve is the temperature in the heating chamber, the green curve is the temperature on the mould surface, the blue curve is the peak internal air temperature (PIAT), and the violet curve is the temperature of the polymer.

Plots in Figure 2a–f show the distributions of temperature as a function of time for brass moulds. Similarly to low-carbon steel moulds, here, one can also observe differences between the temperature on the mould surface, internal air temperature, and the temperature of the polymer. The plots reveal a noticeable difference between the temperature in the heating chamber and the internal air temperature, but the temperature gradient for brass moulds is lower than that obtained for steel moulds. Higher values of the thermo-physical properties of the metallic material reduce the difference between the temperature in the heating chamber and that inside the mould cavity.

Similarly to steel moulds, the temperature of the mould wall and the internal air temperature increase rapidly at the beginning of the process. The temperature increase slows down with the degree of polymer adhesion to the inner surface of the mould cavity and mould wall thickness increase. During the cooling cycle, the temperature on the mould surface and the internal air temperature differ from those observed for steel moulds. With brass moulds, the cooling is faster, which results in a shorter cooling cycle. At first, the cooling of the tool system proceeds rapidly due to a large temperature difference between the mould and the cooling medium. Following the LLDPE’s solidification, the cooling cycle proceeds steadily until the internal air temperature reaches 50 °C.

The peak internal air temperatures were 218.4 °C (M/3/230) and 256.6 °C (M/3/270), while the lowest were 179.8 °C (M/8/230) and 211.8 °C (M/8/270). An increase in the mould wall thickness causes the internal air temperature to decrease by about 5% (3 mm), 12% (5 mm), and 22% (8 mm), relative to the heating chamber temperature, for both 230 °C and 270 °C. The use of brass moulds with a high thermal conductivity coefficient leads to a faster heating rate of the LLDPE powder. The rotational moulding process conducted with brass moulds can result in the production of bubble-free moulded parts. It should be borne in mind that the conditions of rotational moulding affect the transition of the state of aggregation of LLDPE and have an impact on the rate of coalescence of powder particles and diffusion of air bubbles from the molten polymer [37].

The rate of peak internal air temperature increase during the heating cycle for brass moulds with different wall thickness (3, 5, and 8 mm) and heating chamber temperatures (230 °C, 270 °C) was M/3/230—10.92 °C, M/3/270—12.83 °C, M/5/230—10.16 °C, M/5/270—11.94 °C, M/8/230—9 °C, and M/8/270—11 °C.

Depending on the heating chamber temperature and wall thickness, the peak internal air temperature increased by 17.49% (3 mm), 17.52% (5 mm), and 22.22% (8 mm), respectively. The PIAT increase rate is directly related to the heating chamber temperature and mould wall thickness. A higher temperature in the chamber results in a faster PIAT increase rate, and this effect is more noticeable for moulds with thicker walls.

**Figure 3:**
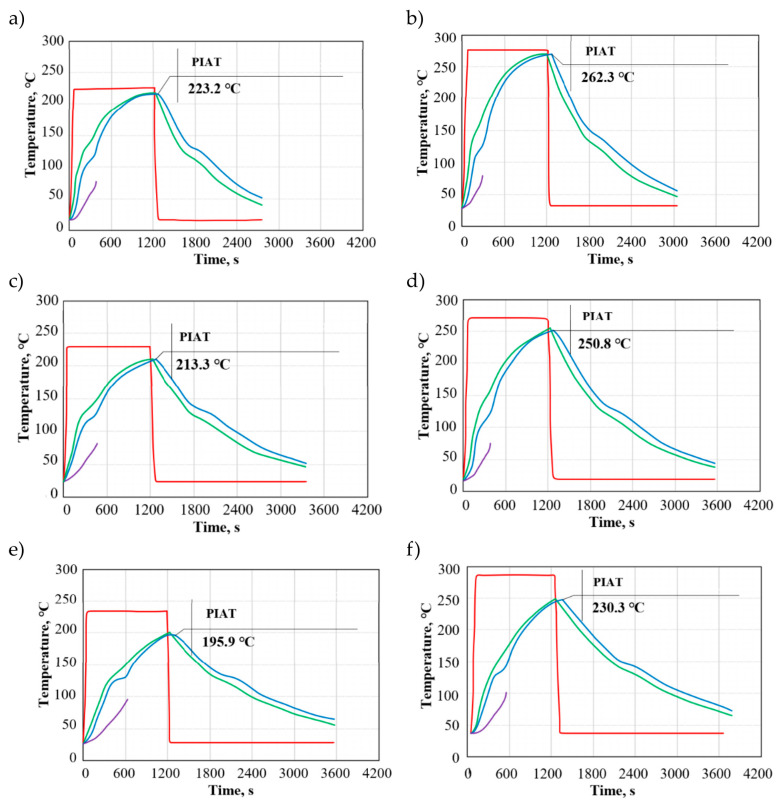
Temperature in a rotational moulding process conducted with aluminium moulds: (**a**) Al/3/230, (**b**) Al/3/270, (**c**) Al/5/230, (**d**) Al/5/270, (**e**) Al/8/230, and (**f**) Al/8/270, where the red curve is the temperature in the heating chamber, the green curve is the temperature on the mould surface, the blue curve is the peak internal air temperature (PIAT), and the violet curve is the temperature of the polymer.

Figure 3a–f shows the temperature as a function of time for aluminium moulds. The results reveal different behaviour patterns depending on the heating chamber temperature and mould wall thickness.

The plots show differences between the temperature in the heating chamber and the internal air temperature; nevertheless, compared to the moulds made of low-carbon steel and brass, the difference is significantly smaller. As previously, one can observe the following relationship: as the mould wall thickness is increased, the difference between the temperature in the heating chamber and the internal air temperature increases despite the lowest temperature gradient value. For a mould with a wall thickness of 3 mm and heating chamber temperatures of 230 °C and 270 °C, this difference is the least noticeable.

At first, the mould wall temperature and the internal air temperature increase rapidly compared to the behaviour observed for low-carbon steel moulds. The cooling is the fastest for aluminium moulds, leading to a considerable reduction in the rotational moulding cycle time.

The peak internal air temperatures were 223.2 °C (Al/3/230) and 262.3 °C (Al/5/270), while the lowest ones were 195.9 °C (Al/8/230) and 230.3 °C (Al/8/270). An increase in mould wall thickness reduced the peak internal air temperature by about 3% (3 mm), 7% (5 mm), and 15% (8 mm) compared to the temperature in the heating chamber at 230 °C and 270 °C alike.

The peak internal air temperature increase for aluminium moulds depending on the mould wall thickness (3 mm, 5 mm, and 8 mm) and heating chamber temperature (230 °C and 270 °C) was as follows: Al/3/230–11.16 °C, Al/3/270–13.11 °C, Al/5/230–10.65 °C, Al/5/270–12.54 °C, Al/8/230—9.79 °C, and Al/8/270—11.51 °C.

P. L. Ramkumar and co-workers [7] demonstrated, using RotoSim simulations, that numerical modelling can reliably reproduce the course of the rotational moulding process in terms of heat transfer behaviour, i.e., predict the temperature evolution over time and identify the key cycle events that are relevant to polymer processing (e.g., reaching PIAT and the time the material spends within critical temperature ranges). In line with this concept, the simulation analysis in the present study enabled a quantitative comparison of the effects of oven temperature and mould parameters on the temperature in the mould cavity. The obtained results indicate that the peak internal air temperature increased by 17.47% (3/230–270), 17.74% (5/230–270), and 17.56% (8/230–270), respectively. As with the brass moulds, it was observed that the peak internal air temperature increase rate for aluminium moulds was higher for a higher temperature in the heating chamber (270 °C) compared to a lower temperature (230 °C). This relationship can be observed for all mould wall thicknesses, which implies that the peak internal air temperature increase is more dependent on the heating chamber temperature rather than on the mould wall thickness. The results showing that increasing the oven temperature leads to a higher maximum temperature (PIAT) and accelerates reaching critical temperatures (thereby shortening part of the heating stage) are consistent with the approach reported in the literature, where PIAT is routinely monitored as a control parameter for the process cycle [38].

The heat transfer coefficient, specific heat, and other thermal properties of the mould are crucial for the determination of the heat transfer rate in rotational moulding, considering interactions between the metallic material and the polymer. It can be observed that among the three study materials, aluminium is characterised by the fastest transfer of heat to the mould cavity. Compared to steel and brass, aluminium has high thermal conductivity, thermal diffusivity, and specific heat.

For all cases, the simulations demonstrated that the difference between the temperature on the mould surface and the peak internal air temperature ranged from 5 °C to 15 °C. The smallest difference between these temperatures occurred at the beginning of the process at 1200 s of the rotational moulding cycle.

**Figure 4:**
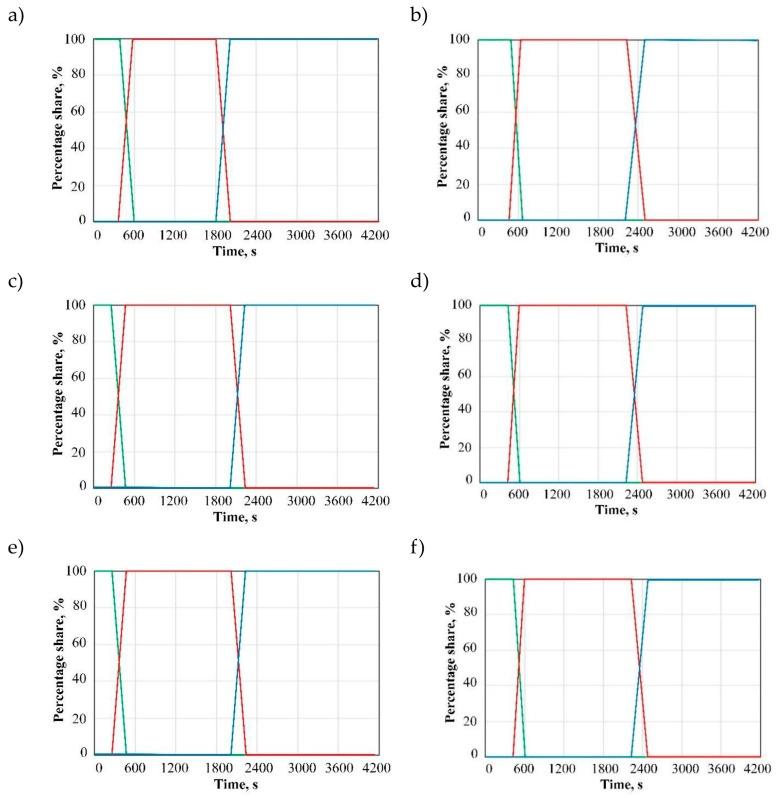
Variations in the states of linear low-density polyethylene in rotational moulding: (**a**) S/3/230, (**b**) S/8/270, (**c**) M/3/230, (**d**) M/8/270, (**e**) Al/3/230, and (**f**) Al/8/270; colours in the plots represent the following: green curve—solid state (for LLDPE in powder form), red curve—plastic/liquid state, and blue curve—solid state.

Another objective of the simulations was to determine the effect of variable factors on the transition of linear low-density polyethylene from a solid to a plastic/liquid state and to a solid state again. Figure 4a–f shows the results obtained for the following samples: S/3/230 (Figure 4a), S/8/270 (Figure 4b), M/5/230 (Figure 4c), M/5/270 (Figure 4d), Al/3/230 (Figure 4e), and Al/8/270 (Figure 4f). For comparison purposes, all plots are made in the same scale. The plots show the percentage share of a given state for LLDPE as a function of the heating and cooling cycle duration. As the polymer powder transforms into a plastic/liquid state, its mass share decreases. After about 1800 s (depending on the case), the molten LLDPE is transformed into a solid state.

**Figure 5:**
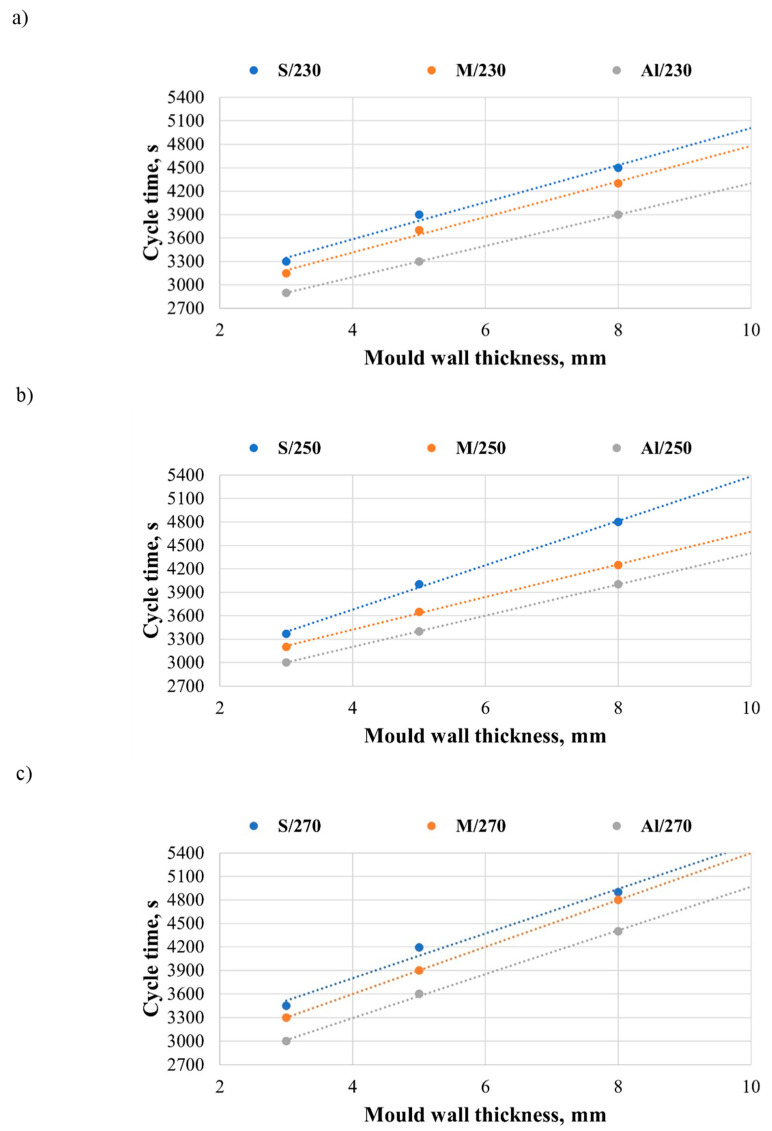
Cycle time of rotational moulding versus mould wall thickness and heating chamber temperature: (**a**) 230 °C, (**b**) 250 °C, and (**c**) 270 °C.

An increase in mould wall thickness maintains the material in a solid state for a longer time. The rate of state transition for LLDPE depends on the heating chamber temperature and mould material. Figure 5a–c shows the effects of mould material, heating zone temperature, and mould wall thickness on the cycle time of rotational moulding. An increase in mould wall thickness causes a decrease in cooling rate. An analysis of the mould material demonstrates that the cycle time is the shortest for aluminium moulds and the longest when low-carbon steel moulds are used.

The cycle time increases with the heating zone temperature (from 230 °C to 270 °C). While the mould heating time remained the same for all analysed configurations of variables, the cooling cycle duration was increased. From an economic viewpoint, a longer mould cooling cycle is unfavourable.

The relationship between mould wall thickness and heating chamber temperature is linear. An increase in mould wall thickness also causes an increase in the cycle time for all analysed cases. The shortest cycle time is observed for the mould with a 3 mm wall thickness, whereas the longest is for those with a wall thickness of 8 mm. For the moulds with a 3 mm wall thickness and a heating temperature of 230 °C, the shortest cycle time was 2900 s (Al/3/230), and the longest was 3300 s (S/3/230). The cycle time of rotational moulding increased by 12.12%.

For the moulds with an 8 mm wall thickness and a heating chamber temperature of 270 °C, the shortest cycle time was 4400 s (Al/8/270), while the longest was 4900 s (S/8/270), i.e., the cycle time increased by 10.20%. The literature emphasises that reducing cycle time is one of the key development directions in rotational moulding due to its impact on energy consumption and overall process efficiency [34]. Increasing the mould wall thickness (from 3 to 8 mm) simultaneously increases the heat conduction path and the tool thermal mass, which results in greater thermal inertia of the system and prolongs the time required to reach characteristic states during the cycle, as well as extending the cooling stage. A similar effect has also been reported in more recent studies, where increasing the mould wall thickness was shown to increase the system’s thermal inertia, extend the time needed to reach PIAT, and affect the internal air temperature evolution [39]. Recent review papers likewise indicate that mould thickness is an important factor that influences the potential for cycle time reduction in rotational moulding [40].

## 4. Discussion

The RotoSim simulation results have led to a number of findings. The numerical results have confirmed that the variable and constant factors of rotational moulding were selected correctly. The numerical results have shown that the tested variables (i.e., mould material, mould wall thickness, and heating chamber temperature) have an impact on the following:

- The distribution of temperature on the mould surface, peak internal air temperature, and the temperature of linear low-density polyethylene.
- Changes in the aggregation states of linear low-density polyethylene.
- The cycle time of rotational moulding.
- Solidification of linear low-density polyethylene.

The use of moulds made of three metallic materials with different wall thicknesses and heating chamber temperatures causes differences in the temperature on the mould surface, internal air temperature, and the temperature of linear low-density polyethylene. For aluminium moulds, the internal air temperature is the most similar to the temperature in the heating chamber. The higher the wall thickness of the mould is, the greater the difference between the internal air temperature and the temperature in the heating chamber becomes. To achieve the internal air temperature that would be close to the heating chamber temperature, the heating cycle duration would have to be increased.

**Figure 6:**
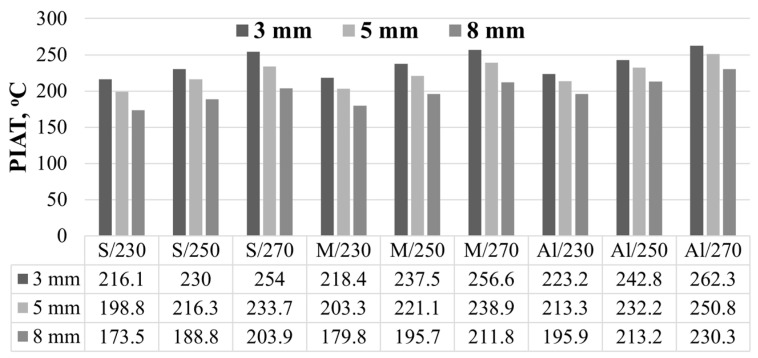
Peak internal air temperature versus mould material, heating chamber temperature, and mould wall thickness.

The numerical RotoSim simulations made it possible to calculate the peak internal air temperature. Figure 6 shows the PIAT values for all cases under analysis. An analysis of the data in the plots and in the table below reveals that for the heating chamber temperature of 230 °C, the peak internal air temperature of a steel mould with a wall thickness of 3 mm is the same (±3 °C) as that observed for a brass mould (M/3/230) and an aluminium mould (Al/5/230).

The peak internal air temperature depends on:

- Thermal properties of the mould material.
- Mould wall thickness.
- The temperature in the heating chamber.

An increase in mould wall thickness impedes the transfer of heat into and out of the mould cavity, as well as leading to a longer cycle time of rotational moulding. Among the three tested metallic materials for moulds, aluminium has the optimal heat transfer properties. An analysis of the cycle times obtained for different mould wall thicknesses reveals that the shortest cycle time is always obtained for an aluminium mould. A reduction in the cycle time of rotational moulding depends on many factors that increase the rate of heat transfer out of and into the mould cavity, that is, on the mould material and mould wall thickness. The higher the heat transfer coefficient is, the shorter the cycle time becomes.

The heating chamber temperature also affects the cycle time of rotational moulding. The higher the temperature in the heating chamber is, the longer the cycle time becomes.

The simulations have shown aluminium to be the optimal material for mould design in rotational moulding. The use of an aluminium mould generates the internal air temperature that is most similar to that in the heating chamber, helps maintain the polymer in a plastic/liquid state for the longest time, and ensures the shortest cycle time. Aluminium moulds exhibit the highest PIAT increase rate at a higher heating chamber temperature (270 °C) compared to a lower temperature (230 °C). In addition, this relationship is observed for all mould wall thicknesses, suggesting that the PIAT increase depends more on the heating chamber temperature rather than on the mould wall thickness (Al/3/230—11.16 °C, Al/3/270—13.11 °C, Al/8/230—9.79 °C, and Al/8/270—11.51 °C).

The RotoSim results provide an in-depth insight into the rotational moulding process. They also make it possible to determine the effect of rotational moulding variables on the properties and characteristics of moulded parts. The knowledge of a theoretical temperature distribution on the mould surface, internal air temperature, and the temperature of the polymer powder material makes it possible to select the optimal heating chamber temperature depending on the mould material and mould wall thickness. The knowledge of a theoretical temperature distribution also makes it possible to determine the cycle time of rotational moulding for specified variable factors. However, when analysing simulation results, one should always bear in mind that the equations describing the effect of variable factors on the rotational moulding process are solved by means of problem-simplifying assumptions, which affect the accuracy of the obtained results.

## 5. Conclusions

The numerical and experimental results of this study lead to the following conclusions and findings:

1. The simulations and experiments of rotational moulding have confirmed that mould material, mould wall thickness, heating chamber temperature, and mould heating time have a significant effect on the stability and cycle time of rotational moulding for linear low-density polyethylene.
2. The simulations of rotational moulding have shown that mould material, wall thickness, heating chamber temperature, and heating time have a significant effect on the distribution of temperature on the mould surface, internal air temperature, LLDPE temperature, and the cycle time of rotational moulding.
3. An increase in the mould wall thickness from 3 to 8 mm impedes the transfer of heat into and out of the mould cavity, as well as increases the cycle time of rotational moulding.
4. The numerical results have shown aluminium to be the optimal material for mould design. The use of an aluminium mould resulted in the internal air temperature distribution being the closest to the heating chamber temperature, the longest time of maintaining the polymer in a plastic/liquid state, and the shortest cycle time.
5. The type of metallic material used for mould fabrication affects mould heating and cooling cycles, with thermal properties of the mould material being of great importance.
6. The appropriate selection of the mould material and its thermal properties allows for shorter cycle times, which translates into reduced thermal energy consumption in the rotational casting process. Therefore, choosing aluminium as a construction material can support the implementation of sustainable production principles.
7. The use of computer simulation tools (RotoSim) during the mould design and process parameter setting stages enables optimised production processes without the need for numerous costly physical tests. This reduces raw material and energy consumption, as well as waste emissions, thus supporting energy and environmental efficiency in production.
8. The presented research results can provide a basis for implementing energy-efficient technological solutions in the plastics processing industry, and are consistent with the concept of green manufacturing while maintaining high product quality and process repeatability.

Taking into account the numerical results regarding the impact of mould material, mould wall thickness, heating chamber temperature, and heating time on the rotational moulding of linear low-density polyethylene (LLDPE), further research will involve experimental validations of these results. The experiments will allow us to assess the degree of agreement between the simulation data and the real process, as well as help determine the impact of selected technological parameters on the quality and efficiency of rotational moulding.

## Figures and Tables

**Table 1:** Thermal properties of materials used in mould design.

Material	Heat Conduction Coefficient, W/(m K)	Specific Heat, J/(kg K)
Copper	386	385
Aluminium (Duralumin)	147	917
Brass	120	400
Carbon steel	51.9	486
Stainless steel	14.5	490
Galvanised steel	52	470
Nickel	21.7	419

**Table 2:** Manufacturer-provided basic specifications of linear low-density polyethylene DOWLEX^®^ 2629UE.

Property	Nominal Value	Particle Structure (Schematically)
Density 23 °C, kg/m^3^ Melt Index (190 °C/2.16 kg), g/10 min Tensile Stress, MPa Tensile Strain, % Flexural Modulus, MPa Shore Hardness, °ShD Heat Deflection Temperature (0.45 MPa), °C Vicat Softening Temperature, °C Melting Temperature, °C Volume Resistance, Ω·m Relative Permittivity (110 Hz)	935 4.0 17.5 650 645 57 65 119 124 1015 2.3	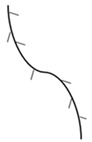

**Table 3:** Chemical composition and properties of mould materials.

Chemical Composition, Properties	Low-Carbon Steel	Brass	Aluminium
	Nominal Value		
Chemical composition, % Yield point, MPa Tensile strength, MPa Vickers hardness, HV10 Density, kg/m^3^ Heat transfer coefficient, W/(m K) Specific heat, J/(kg K) Thermal diffusivity, m^2^/s × 10^−5^	Fe-99.25, C 0.12; Mn 0.6; P 0.045; others: S, Si, P, S, Cr, Mo, Ni, Al., Cu, B, Nb, Co, V, W, Sn, Pb, As, Bi, Ca, Ti, Sb, Zn, Zr ≤ 0.016 140–280 270–410 127.06 7850 31.2 452 0.9	Cu-62.92, Zn-36.96; Fe-0.031; others: Al; Pb, Sn, P, Mn, Ni, Cr, S, Sb, Si ≤ 0.01 130 340 118.93 8440 120 400 3.6	Al-95.64; Mg-3.08; Si-0.34; Fe-0.40; Cu-0.08; Mn-0.024; others: Zn, Ni, V, Pb, Sn, Co, Ag; B, Be, Bi, Ca, Cr, Ga, Li, Na, Sr, Ti, Zr, Cd, In ≤ 0.06 80 240 56.9 2680 168 897 6.9

**Table 4:** PS/DK 3^3^ matrix with decoded variable factors and denotations of samples.

No	Mould Material and Its Heat Transfer Coefficient, W/(m K)	Mould Wall Thickness, mm	Heating Chamber Temperature, °C	Sample Denotation/ Configuration of Variables
1. 2. 3. 4. 5. 6. 7. 8. 9.	Low-carbon steel 31.2	8 8 8 5 5 5 3 3 3	270 250 230 270 250 230 270 250 230	S/8/270 S/8/250 S/8/230 S/5/270 S/5/250 S/5/230 S/3/270 S/3/250 S/3/230
10. 11. 12. 13. 14. 15. 16. 17. 18.	Brass 120	8 8 8 5 5 5 3 3 3	270 250 230 270 250 230 270 250 230	M/8/270 M/8/250 M/8/230 M/5/270 M/5/250 M/5/230 M/3/270 M/3/250 M/3/230
19. 20. 21. 22. 23. 24. 25. 26. 27.	Aluminium 168	8 8 8 5 5 5 3 3 3	270 250 230 270 250 230 270 250 230	Al/8/270 Al/8/250 Al/8/230 Al/5/270 Al/5/250 Al/5/230 Al/3/270 Al/3/250 Al/3/230

In the last column are given the denotations of moulded parts used in the study, e.g., in S/8/270, S—denotes the mould material, 8—is the wall thickness of the mould, and 270—stands for the temperature in the heating zone.

## Data Availability

The original contributions presented in the study are included in the article, further inquiries can be directed to the corresponding author.
